# Correlation of Immunogenicity and Reactogenicity of BNT162b2 and CoronaVac SARS-CoV-2 Vaccines

**DOI:** 10.1128/msphere.00915-21

**Published:** 2022-03-14

**Authors:** Ruiqi Zhang, Ka-Yi Leung, Danlei Liu, Yujing Fan, Lu Lu, Pui-Chun Chan, Kelvin Kai-Wang To, Honglin Chen, Kwok-Yung Yuen, Kwok-Hung Chan, Ivan Fan-Ngai Hung

**Affiliations:** a Department of Medicine, Li Ka Shing Faculty of Medicine, University of Hong Kong, Hong Kong Special Administrative Region, China; b Department of Microbiology, Li Ka Shing Faculty of Medicine, University of Hong Kong, Hong Kong Special Administrative Region, China; c State Key Laboratory for Emerging Infectious Diseases, Li Ka Shing Faculty of Medicine, University of Hong Kong, Hong Kong Special Administrative Region, China; d Carol Yu Centre for Infection, Li Ka Shing Faculty of Medicine, University of Hong Kong, Hong Kong Special Administrative Region, China; Beth Israel Deaconess Medical Center

**Keywords:** BNT162b2, COVID-19, CoronaVac, neutralizing antibodies, side effects, vaccination

## Abstract

COVID-19 infection is a global health issue, and vaccination is the main strategy to control this pandemic. In this study, 189 participants received BNT162b2 or CoronaVac vaccine, and 133 of them recorded adverse events (AEs) daily for 4 weeks after vaccination. Their neutralizing antibody against SARS-CoV-2 was determined with live virus microneutralization (vMN) assay. The vMN geometric mean titer (GMT) on day 56 was 129.9 (95% confidence interval [CI],108.6 to 155.2) in the BNT162b2 group and 13.1 (95% CI, 11.2 to 15.3) in the CoronaVac group. Day 56 vMN GMT was 147.9 (95% CI, 118.9 to 184.1) in females and 129.9 (95% CI, 108.6 to 155.2) in males receiving BNT162b2, while it was 14.0 (95% CI, 11.6 to 17.0) in females and 11.4 (95% CI, 8.7 to 15.0) in males receiving CoronaVac. Injection site pain (88.8%) and redness (77.5%) were the most commonly BNT162b2-related AEs, and injection site pain (37.7%) and tiredness (26.4%) were more frequent in the CoronaVac group. Women showed a higher frequency of headache (45.7% versus 29.4%) and joint pain (26.1 versus 14.7%) than men in BTN162b2 group. Headache (26.5% versus 0%) and tiredness (38.2% versus 5.3%) were more common in women than in men vaccinated with CoronaVac. No correlation between any AE and antibody response was observed in BNT162b2 or CoronaVac platforms. After taking the gender factor into account, in the BNT162b2 group, a low correlation between day 21 vMN titer and redness (rho = 0.34) or itching (rho = 0.32) was presented in females, and a low correlation between day 56 vMN titer and fever (rho = 0.35) was presented in males. Taken together, AEs could have a low correlation with BNT162b2 vaccine response.

**IMPORTANCE** Effective vaccines against SARS-CoV-2 are vital tools for containing the COVID-19 pandemic by increasing population immunity. While currently available vaccines can elicit antibody response against SARS-CoV-2 with high efficacy, the associated side effects may cause vaccine hesitancy. Our work is important in that we have thoroughly analyzed the correlation between immunogenicity and reactogenicity of two COVID-19 vaccines (BNT162b2 and CoronaVac) in the study. Our results showed that women had higher levels of neutralizing antibodies than men after receiving BNT162b2 or CoronaVac. Furthermore, a low correlation was observed between day 21 vMN titer and local reactions (redness and itching) in females, as well as between day 56 vMN titer and fever in males receiving BNT162b2. Thus, common side effects are not always a negative impact of vaccination but may serve as an indicator of immunogenicity of vaccines. Our study may help in increasing the public’s acceptance and confidence over COVID-19 vaccination and ultimately achieving the goal of containing COVID-19 pandemic.

## INTRODUCTION

The coronavirus disease 2019 (COVID-19) pandemic caused by the severe acute respiratory syndrome coronavirus 2 (SARS-CoV-2) has been going on for more than a year and has caused almost 216 million infections and 4.5 million deaths (https://www.who.int/emergencies/diseases/novel-coronavirus-2019). Although some therapies have been confirmed to have partial antiviral effects in clinical trials, there is still a lack of specific treatment (1–3). Relying solely on postinfection treatment cannot reduce the medical burden and infection rate around the world. The clinical symptoms of infected patients range from asymptomatic infection to severe disease, where the presence of the asymptomatic carriers makes the prevention of the pandemic particularly difficult. Therefore, the development of related vaccines and widespread vaccination is considered an effective and promising measure to contain the pandemic.

At present, some vaccines had been evaluated and included in the emergency use list, such as BNT162b2, AZD1222 Vaxzevria, and CoronaVac (https://www.who.int/emergencies/diseases/novel-coronavirus-2019). Among them, BNT162b2 and CoronaVac have been imported by the Hong Kong government in early 2021. BNT162b2 (manufactured by BioNTech and Pfizer) is an RNA-based COVID-19 vaccine which can encode the full-length spike of SARS-CoV-2 to further simulate the complete structure of the virus (4). CoronaVac (Sinovac Life Sciences, Beijing, China) is an inactivated vaccine against SARS-CoV-2 that employs purified inactivated virus to induce specific neutralizing antibodies in the body (5, 6). In phase 3 trials which focused on the safety and effectiveness of the two vaccines, BNT162b2 showed an effectiveness rate of 95%, while that of CoronaVac was 83.5% in preventing COVID-19. The incidence of serious adverse events (AEs) in both vaccines was low, and the most common systemic and local symptoms were tiredness and injection site pain, respectively (4, 7). Age-related studies showed that side effects were milder in the elderly, yet the antibody response was weaker, particularly reflected by the lower levels of neutralizing antibodies and IgG or IgA after the first vaccine dose (4, 8). However, there is currently no study of the relationship between level of effective neutralizing antibodies and the frequency of AEs induced by COVID-19 vaccines.

The aim of this study is to compare the effectiveness and safety of BNT162b2 and CoronaVac vaccines and to explore the relationship between the reactogenicity and immunogenicity of the two vaccines.

## RESULTS

### Subjects.

**FIG 1:**
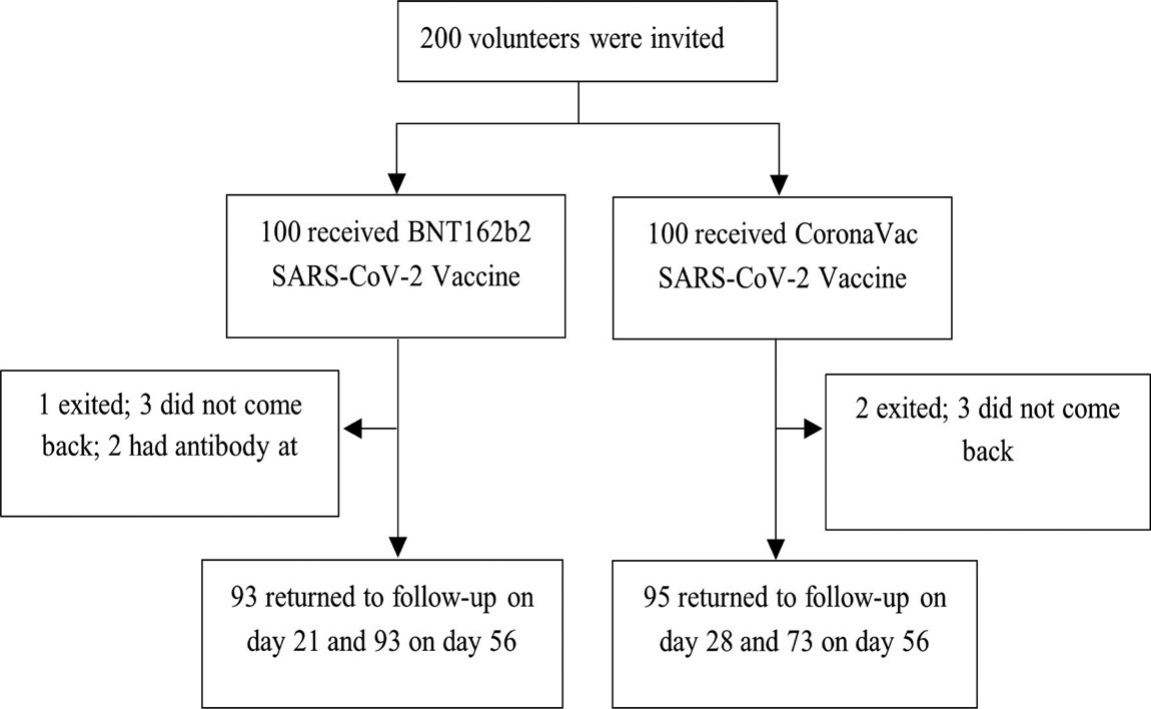
Participant recruitment and research flow diagram.

One hundred and eighty-nine SARS-CoV-2-naive individuals were analyzed in the study, with 94 participants vaccinated with BNT162b2, while 95 received CoronaVac (Fig. 1). In the BNT162b2 group, the median age was 49 years, and 43.6% of participants were men, while the median age was 54 years, and 35.8% of subjects were men in the CoronaVac group (Table 1).

**TABLE 1:** Demographic and clinical baseline characteristics

Characteristic	Data for patients who received:	
	CoronaVac (*n* = 95)	BNT162b2 (*n* = 94)
Median age (range)	54 (20–76)	49 (18–75)
Men (no. [%])	34 (35.8)	41 (43.9)
Women (no. [%])	61 (64.2)	53 (56.1)

### Immunogenicity.

The primer dose of both vaccines induced limited antibody response, with a virus microneutralization (vMN) geometric mean titer (GMT) of 5.7 (95% confidence interval [CI], 5.3 to 6.1) and 11.3 (95% CI, 8.9 to 14.3) in the CoronaVac group and BNT162b2 group, respectively, on day 21 and day 28 post-primer vaccination. On day 56, the vMN GMT was 13.1 (95% CI, 11.2 to 15.3) in CoronaVac group, and 14.9% of females had seroprotection compared with 7.7% of males, while in the BNT162b2 group, the vMN GMT on day 56 was 129.9 (95% CI, 108.6 to 155.2), and 96.7% of participants showed an MN titer of ≥40. Women showed higher vMN GMT (147.9) on day 56 than men (109.3) (Table 2).

**TABLE 2:** Immunogenicity of CoronaVac and BNT162b2^*f*^

Immunogenicity value	Data for patients who received:					
	CoronaVac			BNT162b2		
	Female (*n* = 61)	Male (*n* = 34)	Total (*n* = 95)	Female (*n* = 53)	Male (*n* = 41)	Total (*n* = 94)
Day 0						
GMT value	5.0 (5.0–5.0)	5.0 (5.0–5.0)	5.0 (5.0–5.0)	5.0 (5.0–5.0)	5.0 (5.0–5.0)	5.0 (5.0–5.0)
Day 21/28^*e*^						
GMT value	5.7 (5.2–6.2)	5.8 (5.0–6.6)	5.71 (5.31–6.14)	11.5 (8.4–15.9)^*e*^	10.9 (7.6–15.6)^*e*^	11.3 (8.9–14.3)^*e*^
Seroprotection (no. of patients [%])	0 (0.0)	0 (0.0)	0 (0.0)	10 (18.9)	4 (9.8)	14 (14.9)
Day 56						
GMT value	14.0 (11.6–17.0)	11.4 (8.7–15.0)	13.1 (11.2–15.3)	147.9 (118.9–184.1)	109.3 (81.6–146.3)	129.9 (108.6–155.2)
Seroprotection (no. of patients [%])	7 (14.9)^*a*^	2 (7.7)^*b*^	9 (12.3)	53 (100)^*c*^	36 (90)^*d*^	89 (96.7)

a Forty-seven subjects gave blood on day 56.

b Twenty-six subjects gave blood on day 56.

c Fifty-three subjects gave blood on day 56.

d Forty subjects gave blood on day 56.

e On day 21, patients in the BNT162b2 group received their second dose; those in the CoronaVac group received theirs on day 28. Seroprotection, vMN titer ≥ 40.

f Data are presented as GMT values (95% CI) unless indicated otherwise. GMT, geometric mean titer.

### Safety.

Among these participants, 133, including 80 from the BNT162b2 group and 53 from the CoronaVac group, recorded their symptoms, including systemic reactions (fever, chills, headache, tiredness, and muscle pain) and local reactions (pain, redness, swelling, ecchymosis, and itching) daily after vaccination. In the BNT162b2 group, injection site pain (87.5%), redness (77.5%), tiredness (53.3%), and muscle pain (43.8%) were more frequent than other symptoms (Table 3). For the CoronaVac group, pain at the injection site was reported in 37.7% of participants and tiredness in 26.4%. In the study, women were more likely to experience vaccine-related AEs (Table 3). After vaccination with two doses of the BNT162b2 vaccine, headache (45.7% versus 29.4%), nausea (19.6% versus 8.8%), joint pain (26.1% versus 14.7%), and diarrhea (13.0% versus 2.9%) were more frequent in women than in men (Table 3). In the CoronaVac group, female participants showed higher frequency of headache (26.5% versus 0.0%), tiredness (38.2% versus 5.3%), nausea (11.8% versus 0.0%), and muscle pain (23.5% versus 10.5%) than males (Table 3).

**TABLE 3:** Adverse events after vaccination

Type of reaction	No. (%) of patients with reaction to:					
	CoronaVac			BNT162b2		
	Female (*n* = 34)	Male (*n* = 19)	Total (*n* = 53)	Female (*n* = 46)	Male (*n* = 34)	Total (*n* = 80)
Systemic reactions	19 (55.9)	4 (21.1)	23 (43.4)	34 (73.9)	23 (67.6)	57 (71.3)
Fever	0 (0.0)	0 (0.0)	0 (0.0)	5 (10.9)	4 (11.8)	9 (11.3)
Chills	3 (8.8)	0 (0.0)	3 (5.7)	6 (13.0)	3 (8.8)	9 (11.3)
Headache	9 (26.5)	0 (0.0)	9 (17.0)	21 (45.7)	10 (29.4)	31 (38.8)
Tiredness	13 (38.2)	1 (5.3)	14 (26.4)	27 (58.7)	21 (61.8)	48 (53.3)
Nausea	4 (11.8)	0 (0.0)	4 (7.6)	9 (19.6)	3 (8.8)	12 (15)
Vomiting	1 (2.9)	0 (0.0)	1 (1.9)	1 (2.2)	0 (0.0)	1 (1.3)
Diarrhea	4 (11.8)	1 (5.3)	5 (9.4)	6 (13.0)	1 (2.9)	7 (8.8)
Muscle pain	8 (23.5)	2 (10.5)	10 (18.9)	22 (47.8)	13 (38.2)	35 (43.8)
Joint pain	3 (8.8)	1 (5.3)	4 (7.6)	12 (26.1)	5 (14.7)	17 (21.3)
Skin rash	1 (2.9)	0 (0.0)	1 (1.9)	2 (4.4)	1 (2.9)	3 (3.8)
Local reactions	15 (44.1)	5 (26.3)	20 (37.7)	43 (93.5)	28 (82.4)	71 (88.8)
Pain	15 (44.1)	5 (26.3)	20 (37.7)	42 (91.3)	28 (82.4)	70 (87.5)
Redness	1 (2.9)	0 (0.0)	1 (1.9)	11 (23.9)	7 (20.6)	62 (77.5)
Swelling	1 (2.9)	0 (0.0)	1 (1.9)	17 (37.0)	11 (32.4)	28 (35)
Ecchymosis	0 (0.0)	0 (0.0)	0 (0.0)	5 (10.9)	1 (2.9)	6 (7.5)
Itching	2 (5.9)	0 (0.0)	2 (3.8)	8 (17.4)	5 (14.7)	13 (16.3)

### Correlation between immunogenicity and reactogenicity.

For the BNT162b2 vaccine, no correlation was presented between neutralizing antibody response and any systemic reactions or local reactions (Table 4). Although a poor correlation between swelling (rho = 0.31) or itching (rho = 0.31) and day 28 vMN titer was observed in participants receiving the CoronaVac vaccine, only one and two cases had swelling and itching, respectively (Tables 3 and 4). In the female subgroup receiving the BNT162b2 vaccine, there was a low correlation between day 21 vMN titer and redness at injection site (rho = 0.34) (Table 5). A low correlation (rho = 0.32) between itching and vMN titer on day 21 was also observed. Moreover, in male participants vaccinated with BNT162b2, a low correlation was found between day 56 vMN titer and fever (rho = 0.35) (Table 5). For female and male subgroups of the CoronaVac group, day 28 vMN titer had a poor correlation with swelling (rho = 0.36) and itching (rho = 0.36) in women, while there was a low correlation between diarrhea and day 56 vMN titer (rho = 0.40) in men (Table 5). However, the frequency of swelling (one woman), itching (two women), and diarrhea (one man) induced by the CoronaVac vaccine was low in sex-based subgroups (Table 3).

**TABLE 4:** Correlation between antibody response and adverse events of COVID-19 vaccines

Type of reaction	Rho (*P* value)^*a*^ of MN titer for:			
	CoronaVac (*n* = 53)		BNT162b2 (*n* = 80)	
	Day 28	Day 56	Day 21	Day 56
Systemic reactions	−0.16 (0.257)^*b*^	0.10 (0.467)	0.20 (0.083)	0.008 (0.947)
Fever			0.16 (0.147)	0.22 (0.048)
Chills	−0.062 (0.668)	0.047 (0.737)		0.095 (0.404)
Headache	0.060 (0.678)	0.038 (0.787)	0.000 (0.998)	−0.086 (0.451)
Tiredness	−0.076 (0.600)	0.051 (0.719)	0.14 (0.211)	−0.025 (0.826)
Nausea	−0.089 (0.540)	0.15 (0.271)	−0.058 (0.607)	−0.077 (0.499)
Vomiting		0.21 (0.134)		0.033 (0.771)
Diarrhea	0.18 (0.217)	0.28 (0.039)	0.12 (0.308)	0.13 (0.256)
Muscle pain	−0.059 (0.686)	0.041 (0.772)	0.17 (0.123)	−0.12 (0.282)
Joint pain	−0.089 (0.540)	−0.12 (0.396)	0.12 (0.291)	−0.18 (0.112)
Skin rash	−0.062 (0.668)	−0.062 (0.661)	0.19 (0.101)	0.11 (0.324)
Local reactions	0.14 (0.328)	0.12 (0.400)	0.11 (0.340)	−0.066 (0.565)
Pain	0.14 (0.338)	0.11 (0.417)	0.051 (0.651)	−0.094 (0.411)
Redness		−0.062 (0.661)	0.21 (0.062)	0.036 (0.751)
Swelling	0.31 (0.028)	0.10 (0.458)	0.13 (0.270)	0.077 (0.498)
Ecchymosis			0.17 (0.131)	0.12 (0.278)
Itching	0.31 (0.028)	0.15 (0.287)	0.19 (0.099)	−0.044 (0.703)

a Rho, Spearman’ s correlation coefficient. A value of 0.5 > rho > 0.3 indicates low positive correlation. A *P* value of <0.05 indicates that the correlation coefficient is statistically significant.

b -rho, rho < 0 and indicates negative correlation.

**TABLE 5:** Correlation between antibody response and adverse events of COVID-19 vaccines in females and males

Type of reaction	Rho (*P* value)^*a*^ of MN titer for:							
	CoronaVac (*n* = 53)				BNT162b2 (*n* = 80)			
	Day 28		Day 56		Day 21		Day 56	
	Female (*n* = 34)	Male (*n* = 19)	Female (*n* = 34)	Male (*n* = 19)	Female (*n* = 46)	Male (*n* = 34)	Female (*n* = 46)	Male (*n* = 34)
Systemic reactions	−0.19 (0.281)^*b*^	−0.15 (0.546)	0.12 (0.510)	−0.035 (0.886)	0.25 (0.100)	0.084 (0.638)	0.031 (0.839)	−0.12 (0.517)
Fever					0.20 (0.188)		0.12 (0.414)	0.35 (0.044)
Chills	−0.089 (0.633)		0.016 (0.930)				0.063 (0.678)	0.13 (0.475)
Headache	0.039 (0.834)		−0.023 (0.895)		0.046 (0.764)	−0.159 (0.370)	−0.10 (0.501)	−0.12 (0.514)
Tiredness	−0.12 (0.520)	−0.081 (0.743)	−0.085 (0.634)	0.22 (0.356)	0.11 (0.451)	0.163 (0.357)	0.035 (0.816)	−0.11 (0.530)
Nausea	−0.13 (0.492)		0.16 (0.359)		−0.032 (0.832)	−0.133 (0.455)	−0.048 (0.754)	−0.16 (0.389)
Vomiting			0.26 (0.134)				0.029 (0.849)	
Diarrhea	0.19 (0.300)		0.21 (0.226)	0.40 (0.087)	0.13 (0.381)		0.19 (0.201)	−0.087 (0.631)
Muscle pain	−0.052 (0.779)	−0.12 (0.633)	0.21 (0.227)	−0.42 (0.071)	0.26 (0.077)	−0.062 (0.727)	−0.11 (0.462)	−0.17 (0.334)
Joint pain	−0.089 (0.633)	−0.081 (0.743)	−0.067 (0.707)	−0.29 (0.226)	0.170 (0.261)	−0.022 (0.902)	−0.20 (0.182)	−0.21 (0.248)
Skin rash	−0.089 (0.633)		−0.11 (0.527)		0.21 (0.154)		0.13 (0.387)	0.077 (0.670)
Local reactions	0.12 (0.498)	0.16 (0.521)	0.01 (0.948)	0.27 (0.258)	0.038 (0.800)	0.187 (0.289)	−0.10 (0.501)	−0.050 (0.781)
Pain	0.11 (0.566)	0.16 (0.521)	−0.007 (0.967)	0.27 (0.258)	−0.043 (0.775)	0.188 (0.288)	−0.12 (0.433)	−0.098 (0.586)
Redness			−0.11 (0.527)		0.34 (0.023)	−0.054 (0.762)	0.050 (0.739)	0.016 (0.929)
Swelling	0.36 (0.049)		0.11 (0.527)		0.081 (0.591)	0.15 (0.414)	0.014 (0.925)	0.16 (0.389)
Ecchymosis					0.19 (0.198)		0.20 (0.175)	−0.087 (0.631)
Itching	0.36 (0.049)		0.16 (0.362)		0.32 (0.029)	0.051 (0.775)	−0.023 (0.881)	−0.077 (0.669)

a Rho, Spearman’ s correlation coefficient. A value of 0.5 > rho > 0.3 indicates low positive correlation. A *P* value of <0.05 indicates that the correlation coefficient is statistically significant.

b -rho, rho < 0 and indicates negative correlation.

## DISCUSSION

In the study, the vMN titer against SARS-CoV-2 in sera collected before and after vaccination was compared. It was found that recipients of BNT162b2 had a higher vMN GMT at day 56, and the vMN titer was higher for samples from women than that from men. Regarding side effects, injection site pain and tiredness were common in both vaccine groups. Although no correlation between AE and antibody response was found in the BNT162b2 platform and CoronaVac platform, there was a low correlation between day 21 vMN titer and redness or itching in the subgroup of females vaccinated with BNT162b2.

Some studies have shown that BNT162b2 and CoronaVac vaccines are sufficiently effective, respectively, conferring 95% and 83.5% protection against COVID-19 (4, 7). Our study further compared the two vaccines and found that BNT162b2 elicited higher vMN GMT than CoronaVac after the second dose (129.9 versus 13.1; *P < *0.0001). One study has reported similar disparity in neutralizing antibody responses induced by the two vaccines (9). However, in another study conducted by Wu et al., the day 56 vMN GMT in subjects receiving two doses of CoronaVac is 64.4 (95% CI, 41.5 to 99.7) (10), which is higher than that found in our study (day 56 vMN GMT, 13.1 [95% CI, 11.2 to 15.3]). Such difference may be caused by different procedures of vMN assay. Wu et al. incubated virus-serum mixture with suspended Vero cells and observed a cytopathic effect (CPE) on day 5 (10). In our study, the VeroE6 TMPRSS2 cell line was used and cultured on multiwell plates 1 day before the addition of virus-serum mixture, and then a CPE was observed on day 3.

Moreover, we found that women showed higher levels of neutralizing antibodies than men in both vaccine groups, though the difference is not statistically significant. There have been some related studies suggesting that gender factors can cause immune responses of different intensities. Zeng et al. found that female patients show higher level of IgG antibody against SARS-CoV-2 than males (11). The immune response of women after being vaccinated with seasonal influenza vaccine is also more pronounced than that of men (12). The reason may be that the absolute number of CD4-positive (CD4^+^) lymphocytes in women is higher than that in men, leading to an increased immune response. Meanwhile, a comparison of cytokine production under immune conditions showed a greater production of TH1 cytokines in females (13, 14). Therefore, the result of females having higher postvaccination neutralizing antibody levels than males from this study may be related to these factors.

BNT162b2-related AEs, including injection site pain, headache, fatigue, muscle pain, chills, and fever, have been reported (15), and around 10% to 20% of recipients of CoronaVac vaccine show injection site pain and tiredness (6). In our study, the frequency of redness and swelling induced by BNT162b2 was 77.5% and 35%, respectively, while a lower frequency (redness, 6%; swelling, 7%) was reported by Polack et al. (4). The reasons for such discrepancy may be as follows. First, the adverse events in our study were recorded for 4 weeks after each dose of vaccine, which were longer than 7 days in that study. Second, the difference in nurses’ injection technique would affect the frequency and severity of local reactions when administrating vaccine. Third, the symptom diary was completed by vaccinees in the study, and individual difference could also lead to the variation in local reaction frequency between the two studies.

In the subgroup analysis of gender, women not only mounted stronger antibody response but also experienced more frequent AEs, including headache, nausea, muscle pain, joint pain, and injection site pain than men after vaccination with BNT162b2 (Table 3). In the CoronaVac group, headache, tiredness, muscle pain, and injection site pain were more prevalent in females than in males (Table 3). The different levels of vaccine-related AEs between women and men have also been reported by other studies (16, 17). A clinical study has pointed out that female participants receiving the BNT162b2 vaccine suffer from more frequent local and systemic vaccination reactions than men (16). In another trial of the CoronaVac vaccine in Turkey, injection site pain, headache, fatigue, and muscle pain are more common in females than males (17). As women are more likely to have stronger immune responses than men after vaccination (12), we infer that such difference may be related to the more frequent AEs women experienced. Here, a low correlation between vMN titer on day 21 and injection site reactions (redness, rho = 0.336; itching, rho = 0.321) was observed in female subjects from the BNT162b2 group. Interestingly, in the male group of BNT162b2, we also found an association between fever and vMN titer on day 56 (rho = 0.353). This suggests that adverse reactions may be related to a more robust immune response after vaccination with BNT162b2. In a trial conducted in 2020, recipients with high dose of COVID-19 vaccine had higher levels of neutralizing antibody (GMT, 297), of whom 25% suffered injection site reaction, while only 4.2% of recipients with a medium dose of vaccine (GMT, 206) had injection site reaction (18). Besides COVID-19 vaccines, such a correlation between AE and immunogenicity in influenza vaccines has also been found. In our previous clinical study, subjects receiving intradermal influenza vaccine adjuvanted with topical imiquimod showed significantly higher levels of neutralizing antibody on day 21 (*P < *0.0001) and higher frequency of redness (*P < *0.0001) and swelling (*P = *0.001) than those receiving intradermal vaccine with topical aqueous cream (19). In 2020, it has been reported that postvaccination feverishness is associated with higher mean fold rise in antibody titers against different vaccine strains, especially significant for influenza A H1N1 (20).

Moreover, a correlation between BNT162b2 vaccine-induced inflammatory cytokines and antibody levels suggested by a recent study (21) further solidifies our hypothesis on the association between vaccine reactogenicity and immunogenicity. The release of inflammatory mediators, including chemokines and cytokines, is part of the complex series of innate immune events induced upon vaccination. This is essential for eliciting antigen-specific immune responses, which is necessary for providing protection. The resulted inflammatory events could cause the development of symptoms of injection site inflammation (e.g., pain and redness), while the circulated inflammation products could further cause systemic side effects (e.g., fever and fatigue) (22). It is reported that there was a highly significant increase of interleukin 6 (IL-6) at day 1 after both the 1st (∼1.5×) and the 2nd dose (∼2×) of BNT162b2 mRNA vaccine in naive vaccinees (21), while tumor necrosis factor alpha (TNF-α) also showed a significant increase after the 2nd vaccination (21). Positive correlations were also identified between anti-spike-RBD antibody level at peak (∼day 36) and the increase in IL-15, interferon gamma (IFN-γ), chemokines macrophage inflammatory protein (MIP)-1α/CCL3 and MIP-1β/CCL4, and inflammatory markers IL-12 and IL-23p40 upon the 2nd dose of vaccine (21). All these cytokines and chemokines which are involved in inflammatory reactions support the role of leukocyte recruitment in the priming of humoral responses as well as the shaping of adaptive immunity. Hence, the association between postvaccination inflammatory mediators and antigen-specific antibody level further supports the correlation between vaccine reactogenicity and immunogenicity.

Our study showed the vaccines were effective and that a correlation between immune response and AEs of BNT162b2 vaccine was present. Nevertheless, the small sample size in this study was one of the limitations. Further studies should be done to confirm the correlation between antibody response and side effects of COVID-19 vaccine. Meanwhile, the symptom diaries filled out by the participants after vaccination were not fully returned, and subjective factors of participants were inevitably present in the severity score. Therefore, result bias cannot be ruled out in the correlation analysis.

In conclusion, low correlations between antibody titer and symptoms in both genders were found. The degree of AEs would cause different negative impacts on the public’s acceptance of vaccination and, hence, the progress of the vaccination program. However, AEs may work as an indicator of the immunogenicity of the vaccine. Furthermore, a higher dose of vaccine for men may be considered so as to enhance antibody response to a level that is comparable with women.

## MATERIALS AND METHODS

### Study design and participants.

This study was an open trial conducted at two vaccination centers in Hong Kong. Adults aged 18 to 76 years who met the vaccination indications were recruited and screened. Patients with a history of SARS-CoV-2 infection and those who had not recovered were all excluded. We had obtained informed consent from the participants. The study was approved by the institutional review board of the University of Hong Kong and Hospital Authority of Hong Kong.

### Procedures.

Participants in the study had the right to choose between BNT162b2 and CoronaVac for vaccination. They received the primer and booster doses of vaccine on day 0 and day 21 in the BNT162b2 group, while subjects in the CoronaVac group were vaccinated on day 0 and day 28. There was no placebo group in the study. Nurses asked the participants about their physical condition before vaccination and informed them of the vaccination risks and precautions. All participants were given a symptom diary to record any systemic and local AEs for 4 weeks after each dose.

Blood was collected from the participants for antibody assay at three time points, including baseline, day 21 (BNT162b2) or day 28 (CoronaVac), and day 56. Live virus microneutralization (vMN) assay described previously (23) was used to measure serum-neutralizing antibody titer. VeroE6 TMPRSS2 cells were seeded in a 96-well plate and incubated at 37°C in 5% CO_2_. The serum samples were heat inactivated at 56°C for 30 min and were prepared for vMN assay by a 5-fold dilution first and then 2-fold serial dilutions. One hundred microliters of 50% tissue culture infective dose (TCID_50_) of SARS-CoV-2 virus was mixed with the diluted serum at a 1:1 ratio and incubated at 37°C for 1.5 h, and then added to the cells and placed in a 37°C incubator again for 72 h. Cytopathic effect (CPE) was observed and recorded at 72 h postinfection. The titer of vMN was defined as the maximum dilution of serum at which the percentage of CPE is equal to or less than 50%. Experiments with the live virus were performed in a biosafety level 3 facility.

### Outcomes.

The primary outcome was seroprotection after vaccination. Secondary outcomes were the incidence of AE and the correlation between immune response and side effects. Symptom data were obtained from the diary filled out by the subjects after vaccination. Systemic symptoms included fever, chills, headache, tiredness, nausea, vomit, diarrhea, muscle pain, joint pain, and skin rash. Injection site pain, redness, swelling, ecchymoses, and itching were listed as local symptoms. Fever in systematic symptoms was divided into 4 grades, grade 1 from 38.0°C to 38.4°C, grade 2 from 38.5°C to 38.9°C, grade 3 from 39.0°C to 40.0°C, and grade 4 was over 40.0°C. For local symptoms, pain was graded in 3 levels; grade 1 was pain when being touched, grade 2 was pain when raising the hand, and grade 3 was pain with any movement. Redness, swelling, and ecchymoses were based on the same standard; grade 1 ranged from 2 cm to 5 cm, grade 2 from 5 cm to 10 cm, and grade 3 was over 10 cm.

### Statistical analysis.

Statistical inference of normally distributed continuous variables was performed using *t* tests, including demographic parameters, seroprotection, and symptom duration. The chi-square test was used for categorical data. The association between antibody response and AE was analyzed by multivariate analysis. SPSS Statistics 27.0 was used for statistical computation. Results are considered significant at a *P* value of <0.05.
